# Impact of CYP1A2 Genotypes on the Ergogenic Effects and Subjective Mood States of Caffeine Ingestion in Resistance-Trained Women

**DOI:** 10.3390/nu16162767

**Published:** 2024-08-20

**Authors:** Jessica M. Prather, Christine M. Florez, Amie Vargas, Bella Soto, Audrey Ross, Abby Harrison, Ariane H. Secrest, Darryn S. Willoughby, Sydney Kutter, Lem W. Taylor

**Affiliations:** 1 Department of Health and Human Performance, Concordia University Chicago, River Forest, IL 60305, USA; 2 Energy Balance & Body Composition Laboratory, Department of Kinesiology & Sport Management, Texas Tech University, Lubbock, TX 79409, USA; 3 Human Performance Lab, School of Exercise & Sport Science, University of Mary Hardin-Baylor, Belton, TX 76513, USA; 4 Public Health Program, School of Health Professions, University of Mary Hardin-Baylor, Belton, TX 76513, USA; 5 Physician Assistant Program, School of Health Professions, University of Mary Hardin-Baylor, Belton, TX 76513, USA; 6 School of Medicine, Baylor College of Medicine, Temple, TX 76508, USA; 7 Physical Therapy Program, School of Health Professions, University of Mary Hardin-Baylor, Belton, TX 76513, USA

**Keywords:** caffeine, CYP1A2, resistance training, females

## Abstract

Caffeine’s metabolism is determined by CYP1A2 genotypes: AC/CC (SLOW) and AA (FAST). This trial evaluated CYP1A2 genotypes’ impact on exercise and cognitive effects in 36 resistance-trained females assessed under placebo (PL) and caffeine (6 mg/kg bw anhydrous caffeine-CAF) conditions, before ingestion and throughout the session. 23andMe^®^ (San Francisco, CA, USA) determined genotypes using saliva. Data were analyzed using two-way RMANOVA and paired-samples *t*-tests (*p* < 0.05). A significant main effect for genotype existed for leg press repetitions to failure (RTF) for CAF (*p* = 0.038), with the FAST group performing more repetitions than the SLOW (*p* = 0.027). There was a significant condition x genotype interaction for the subjective outcome index score (*p* = 0.045), with significant differences for time (*p* < 0.01) and between genotype (*p* < 0.001). Follow-up analysis revealed a higher total score (*p* = 0.028) following CAF for the FAST group and a lower total score (*p* < 0.01) in the SLOW group. Dizziness was reported following CAF in the SLOW group (*p* = 0.014; Cohen’s *d* = 0.725). Aside from leg press RTF, subjective outcome index score, and dizziness, the genotype groups experienced similar responses to resistance exercise performance and subjective mood states following caffeine ingestion.

## 1. Introduction

Caffeine (1,3,7-trimethylxanthine) is a neurostimulator that is one of the most consumed drugs in the world. After it is metabolized in the liver by the cytochrome p450 1A2 (CYP1A2) enzyme, evidence indicates that it has a considerable effect on hormonal, metabolic, muscular, cardiovascular, pulmonary, and renal function [1]. This enzyme is encoded by the CYP1A2 gene, from which a single nucleotide polymorphism (SNP), rs762551, impacts the speed that caffeine is metabolized [2]. This SNP is used to determine whether an individual is a slow metabolizer with the heterozygous AC or homozygous CC genotype (SLOW), or a fast metabolizer with the homozygous AA genotype (FAST) [2]. The FAST and SLOW groupings are determined based on the rate at which they metabolize caffeine [3]. Caffeine is often used as an ergogenic aid; one of the proposed mechanisms of its effect regarding performance is due to the binding of caffeine, as well as certain caffeine metabolites, to adenosine receptors throughout the body due to the similar molecular structure [3]. When caffeine binds to the adenosine receptors, it blocks adenosine’s ability to reduce nerve activity, resulting in an increased excitatory state.

Regarding pharmacokinetics, absorption via the gastrointestinal tract is typically complete by 60 min post-ingestion, with 99% of the substance absorbed after approximately 45 min [4]; however, peak plasma concentration is typically observed anywhere from 15 to 120 min, with a half-life of 1.5–10 h [4]. The half-life duration and clearance of caffeine and its metabolites may be impacted by various external factors such as high and frequent dosing and habituation, genetics, the menstrual cycle, and other drugs that are taken concurrently. Research to date shows acute ergogenic effects of caffeine at a wide range of doses including generalized recommendations of 3–6 mg/kg bw of caffeine, consumed approximately 60 min prior to exercise [5]. Although there are numerous studies that have examined this and support the fact that caffeine provides an ergogenic effect on performance in both trained and untrained males and females [4], there is limited research on the degree of interindividual variability and where those differences stem from. For example, the menstrual cycle impacts CYP1A2 activity, potentially due to the proximity of the start of menstruation and fluctuating progesterone levels, thus resulting in slower systemic clearance of caffeine during the luteal phase when compared to the follicular phase; yet, half-life does not appear to be affected [6]. Variability in the metabolism of caffeine may also stem from the use of oral contraceptives in women, as it has been shown to reduce the metabolic rate and activity of CYP1A2 [7]. Habitual intake also impacts caffeine’s effects as it can increase tolerance to known side effects [8], leading to a physiological (i.e., blood pressure) adjustment with chronic consumption. However, the impact of habituation on the performance-enhancing effect of caffeine is still unclear.

In recent years, exploration of the role that one’s genetics play in moderating the degree to which caffeine enhances performance and its interindividual variability has gained popularity. Research that discusses the role that the various CYP1A2 genotypes play on the performance-enhancing effect of caffeine in relation to exercise is rising, but contradictory in nature [1,2,9,10,11,12,13,14,15,16]. While some research supports the notion that CYP1A2 genotypes impact exercise performance [2,9,10,12,15,16], there are others that fail to corroborate these findings [1,11,13,14]. Research that is solely focused on the CYP1A2 genotype’s impact on anaerobic resistance training in a mixed population is scarce [1,2,14,15,16]. There are a few studies that have shown little to no differences between CYP1A2 genotypes in exercise performance after ingesting caffeine at varying doses [1,14,15,16]. At the time of this literature search for relevant studies, there is only one reported study in which participants with the SLOW genotype had a greater improvement in performance than the FAST genotype after ingesting caffeine and rinsing their mouth with a caffeine solution [12]; however, this was based on a 3 km cycling time trial. Furthermore, there have been no reported findings on the influence of the different CYP1A2 genotypes on the ergogenic effects of caffeine in the female-only population following a resistance training protocol [17]. Therefore, this is currently the first and only study that focuses on a female-only population while also accounting for menstrual cycle phases. By choosing to use only females, instead of a mixed population, the control of biological differences between genders is better accounted for and potential confounding variables are limited. Thus, the differences between genotypes and the influence they have on caffeine-induced performance enhancements are better able to be understood.

Despite some exploration of CYP1A2 genotypes and caffeine’s performance benefits in anaerobic and resistance training exercise, current data are limited and inconsistent. Although male and mixed populations are represented in the current literature, there have been no reported findings in female-only populations at the time of this literature search [17]. Therefore, the purpose of this experiment was to investigate the impact that CYP1A2 genotypes have on the ergogenic and cognitive effects of caffeine in females, with a focus on resistance training.

## 2. Materials and Methods

### 2.1. Participants

Participants in this study consisted of 36 (FAST, n = 20, 21.6 ± 2.2 y, 163.2 ± 9.6 cm, 70.3 ± 15.4 kg; SLOW, n = 16, 22.3 ± 3.8 y, 165.6 ± 5.3 cm, 68.7 ± 14.3 kg) healthy, resistance-trained female college students who habitually consumed caffeine and were not on any form of birth control. Participants were required to have a consistent history of resistance training at least 2–3 times per week for a minimum of one year and were required to be regular caffeine users (75–200 mg/day). Written and dated informed consent was obtained from each participant, and approval was granted for this study by the University of Mary Hardin-Baylor Institutional Review Board. Participants agreed to and were reminded to refrain from exercise 24 h prior to each testing visit. Participants were given a last meal time the evening before to standardize a 12 h fasting period before each testing visit. Participants with sensitivity issues following caffeine ingestion were excluded. Any participant reporting unusual adverse events associated with the study, as determined in consultation with the study investigators or their doctor, was removed from participation.

### 2.2. Experimental Design

**Figure 1:**
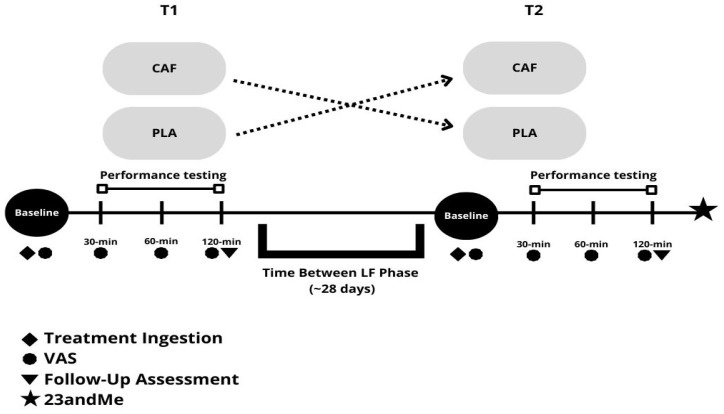
Overview of treatment protocol.

A convenience sample recruited via flyers, social media, and word of mouth was used for this double-blind, placebo-controlled, crossover study (Figure 1). Randomization was achieved using an online random number generator (random.org) prior to the first testing session by a single staff member who prepared the treatments for ingestion and transferred this to a staff member that executed the resistance exercise session to remain blind from the staff and participant. Participants were verbally instructed on the methods of the study by Human Performance Lab staff at the University of Mary Hardin-Baylor and then performed a familiarization session where they completed a medical history form, a general health and information screening form, a physical activity readiness questionnaire, and a caffeine consumption questionnaire. Participants‘ height and weight were measured for the Dual-Energy X-Ray Absorptiometry (DXA) and InBody Bioelectrical Impedance Analysis body composition tests.

#### Testing Protocol

This protocol included a familiarization session followed by two subsequent testing sessions. The familiarization testing session consisted of one-repetition maximum (1RM) baseline testing for both the leg press and bench press exercises, followed by a Wingate cycle test where participants were fitted for seat height and handlebar settings. Testing for 1RM was conducted in accordance with the National Strength and Conditioning Association (NSCA) guidelines after a 5 min warmup on a stationary bike. The leg press 1RM protocol began with warmup sets performed at 50% and 70% of predicted 1RM with 2 min of rest in between. The weight on leg press increased 10–20% after each successful repetition until volitional failure, poor technique, or the participant chose not to continue. Participants were allowed a 3 min rest after each attempt with a maximum of 5 attempts. Following a 5 min rest, 1RM for bench press was established using the same protocol. After a bench press 1RM was established, participants rested for 5 min and completed the Wingate cycle test. Participants performed the Wingate protocol on a Wattbike PRO (Bridgford, Nottingham, UK) cycle ergometer using the bike setup measurements established in familiarization. Following a 90 s warmup at 60 RPM, participants were given a 10 s countdown and verbally instructed to maximize RPM prior to the start of the test. The air resistance setting was increased according to manufacturer (7.5% of body mass for resistance with sample rate of 100 times per second) guidelines and participants cycled with all-out effort for the 30 s test to complete the experimental exercise testing session. Participants were then scheduled for testing to be completed during the late follicular phase (approximately days 6–14) of their menstrual cycle for both subsequent testing sessions, which were approximately 28 days apart.

Testing session one (T1) and testing session two (T2) protocols were identical. Body weight, heart rate, and blood pressure measurements were assessed at baseline. This was followed by the consumption of their randomly assigned treatment (6 milligrams per kilogram of body weight of powdered caffeine anhydrous mixed in 20 oz of Propel-CAF or 20 oz of Propel as placebo-PLA) for their respective testing session. Thirty minutes after consumption, participants began the leg press 1RM and repetitions to failure (RTF) and the bench press 1RM and RTF, followed by the Wingate cycle test for mean and peak power using standardized rest periods between attempts and tests used in the familiarization session. Participants filled out a Visual Analog Scale (VAS) questionnaire that assessed mood states and energy levels (alertness, concentration, energy, fatigue, and focus) pre-ingestion and again at 30, 60, and 120 min post-treatment ingestion. These items were summed to create a composite subjective outcome index score, a well-established practice for single variable analysis of perceived measures in various fields [18,19], including studies assessing subjective effects of caffeine [20]. At the end of each testing session, participants filled out a side effects follow-up questionnaire where they were able to record any adverse side effects (including dizziness, headache, fast or racing heart rate, heart skipping or palpitations, shortness of breath, nervousness, and blurred vision) and the severity of each on a scale of 0–5 (0 = none; 1 = minimal; 2 = slight; 3 = moderate; 4 = severe; 5 = very severe). At the end of the second testing session, the participants provided a saliva sample via a 23andMe^®^ collection kit. Prior to T1 and T2, participants were instructed to fast for 12 h and refrain from caffeine consumption and any vigorous exercise for at least 24 h prior to or at any time during the testing session.

### 2.3. Genotype Analysis

Genotype analysis kits were purchased from 23andMe^®^, a company that uses genotyping to detect over 150 variants in the genomic DNA from one’s saliva. Participants were instructed to provide a saliva sample for a 23andMe^®^ kit provided by the Human Performance Lab and were sent off for analysis after the completion of both T1 and T2. The samples were then sent to the designated address for genotyping via 23andMe^®^. Only the specific information about the CYP1A2 genotypes relevant to this study was recorded and reported. The SNP rs62551 was obtained from the raw 23andMe DNA data, which provided participants’ respective genotypes (AA: n = 20; AC: n = 12; CC: n = 4). Due to uneven sample sizes and based on the premise that C allele carriers are designated slow metabolizers, these were split into their respective FAST and SLOW metabolizing groups for statistical analysis.

### 2.4. Statistical Design

The sample size for this study was determined by using the G*Power (version 3 for Windows) application [21] to perform a power analysis with a power of 0.8 and a medium effect size of 0.5. For this experimental design, two genotype groups and two treatment groups are used; thus, a sample size of 42 was revealed as adequate to provide sufficient power for the statistical analysis. Participant characteristics (age, height, weight, lean mass, body fat mass, and percent body fat) were reported using descriptive statistics. Subjective outcome data were analyzed using a two-way analysis of variance (ANOVA) with repeated measures. One-repetition maximum (1RM), repetitions to failure (RTF), and Wingate mean and peak power at each testing session were compared using a two-way ANOVA with repeated measures for genotype group (FAST vs. SLOW) as a between-participants factor, and condition (CAF, PLA) as a within-participant variable. Follow-up assessment data were analyzed using a paired-samples *t*-test with Cohen’s d effect sizes. All statistical analyses were performed using IBM SPSS Statistics 25 (IBM, Armonk, NY, USA). Data are presented as means ± standard deviation and statistical significance was set a priori at *p* < 0.05.

## 3. Results

### 3.1. Participant Characteristics

Of the 43 participants that volunteered for this study, 36 were able to complete all three testing sessions (see Table 1 for descriptive data). The seven participants that were unable to complete the study exercised their right to withdraw due to scheduling conflicts (n = 3), loss of interest (n = 3), or hypersensitivity to caffeine (n = 1). Participant body weight from did not change (*p* = 0.578) from T1 to T2.

### 3.2. Exercise Performance

#### 3.2.1. Maximum Strength (1RM)

**Figure 2:**
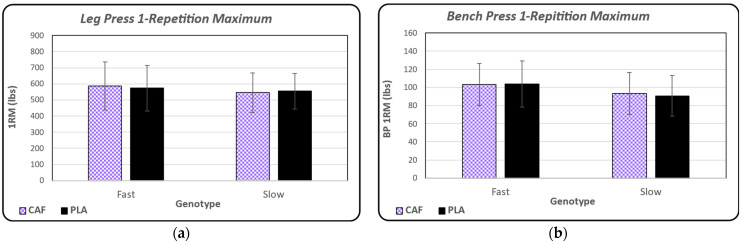
(**a**) Mean ± standard deviation values for leg press max in both conditions (CAF vs. PLA) and genotype groups (FAST vs. SLOW). There were no observed statistical differences between treatment or genotype groups. (**b**) Mean ± standard deviation values for bench press 1RM in both conditions (CAF vs. PLA) and genotype groups (FAST vs. SLOW). There were no observed statistical differences between treatment or genotype groups.

Leg press 1RM for both conditions is shown in Figure 2a and is separated by genotype group. Statistical analyses revealed that there were no significant main effects observed between conditions (*p* = 0.852) or genotype (*p* = 0.254). Bench press 1RM for both conditions, also separated by genotype group, is shown in Figure 2b. Similar to leg press 1RM, there were no significant main effects observed between conditions (*p* = 0.307) or genotypes (*p* = 0.214).

#### 3.2.2. Muscular Endurance (RTF)

**Figure 3:**
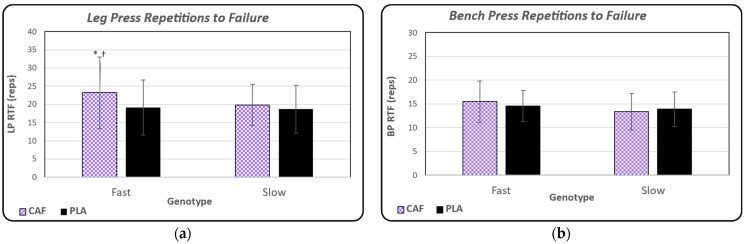
(**a**) Mean ± standard deviation values for the total number of RTF performed during the leg press exercise in both conditions (CAF vs. PLA) and genotype groups (FAST, SLOW). * denotes significant difference between conditions (CAF vs. PLA) in the FAST group where *p* = 0.038; † denotes significant difference between genotype groups (FAST vs. SLOW) where *p* = 0.027. (**b**) Mean ± standard deviation values for the total number of bench press repetitions to failure in both conditions (CAF vs. PLA) and genotype groups (FAST vs. SLOW). There were no observed statistical differences between treatment or genotype groups.

Leg press RTF performance for both conditions is shown in Figure 3a, separated by genotype group. There was an observed main effect between treatment groups for leg press RTF (*p* = 0.038). Within the FAST group, post hoc analysis revealed the participants performed more RTF with the CAF condition than PLA (*p* = 0.027). In contrast, for bench press RTF (Figure 3b), there were no observed differences between conditions (*p* = 0.783) or between genotypes (*p* = 0.339).

#### 3.2.3. Anaerobic Power

**Figure 4:**
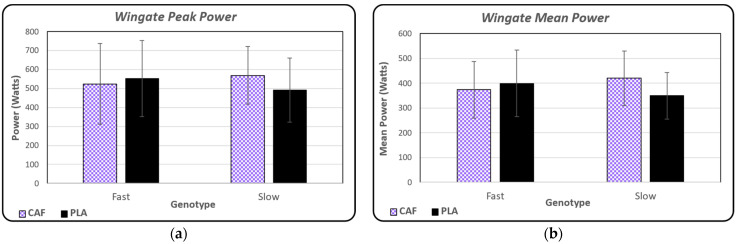
(**a**) Mean ± standard deviation values for peak power output in watts from the Wingate cycle test in both conditions (CAF vs. PLA) and genotype groups (FAST vs. SLOW). There were no observed statistical differences between treatment or genotype groups. (**b**) Mean ± standard deviation values for mean power output in watts from the Wingate cycle test in both conditions (CAF vs. PLA) and genotype groups (FAST vs. SLOW). There were no observed statistical differences between treatment or genotype groups.

The Wingate peak power performance is shown for both conditions in Figure 4a, separated by genotype group. Statistical analyses revealed no significant differences between conditions (*p* = 0.593) or between genotype groups (*p* = 0.246). Wingate mean power performance is shown for both conditions in Figure 4b, also separated by genotype group. Despite a higher mean power for the CAF group, there were no observed main effects of significant differences between conditions (*p* = 0.412) or genotype groups (*p* = 0.093).

#### 3.2.4. Subjective Outcomes

**Figure 5:**
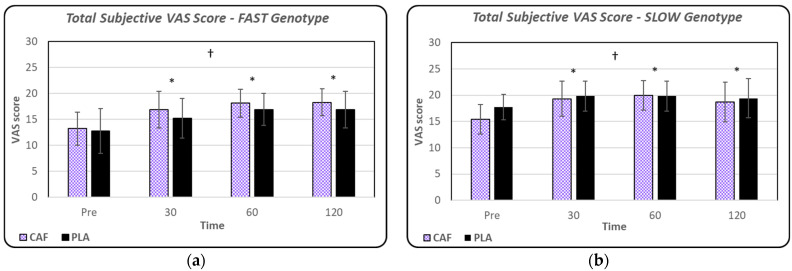
(**a**) Mean ± standard deviation values for subjective outcome index score for FAST (CAF vs. PLA). (**b**) Mean ± standard deviation values for subjective outcome index score for SLOW (CAF vs. PLA). * denotes significant main effect for time (*p* < 0.01); † denotes significant difference between genotype groups (FAST vs. SLOW).

The total subjective VAS scores (combined subjective outcome index score) are depicted in Figure 5a,b. A significant treatment-by-genotype interaction was observed in the subjective outcome index score (F = 4.37; ή^2^ = 0.124; *p* = 0.045). Additionally, the two-way ANOVA revealed significant main effects for time (*p* < 0.01) and genotype (*p* < 0.001). Follow-up analysis revealed that the FAST group had a higher total subjective outcome index score in the CAF group versus the PLA group (*p* = 0.028), while the SLOW group had a lower total subjective outcome index score (*p* < 0.01).

A side effects follow-up assessment was given to the participants to complete after each testing session to record side effects and the severity each, if any at all (Table 2). The analysis revealed that approximately two hours after ingestion of caffeine, the subjects experienced more dizziness than after ingestion of the placebo (*p* = 0.003). Further, a paired *t*-test between conditions for dizziness revealed significant differences between conditions in the slow-metabolizing group, with a medium to large effect size (*p* = 0.014; Cohen’s *d* = 0.725). No other side effect variables assessed were significantly different between CAF and PL.

## 4. Discussion

In this study, we assessed the impact of CYP1A2 genotypes on the ergogenic and cognitive effects of caffeine in a resistance-trained female population with a moderate dose of caffeine (6 mg/kg bw). To our knowledge, this is the first study to examine the effects of CYP1A2 genotypes on caffeine in a female resistance-trained population.

Muscular strength was not impacted by caffeine ingestion or influenced by genotype condition. In similar previous research, two groups [15,22] assessed isometric handgrip strength in trained males and females with a handgrip dynamometer. Munoz and colleagues [15] administered a 3 mg/kg bw dose of caffeine or placebo, while Spineli and researchers [22] administered a 6 mg/kg bw dose. Each of these studies reported that there was neither a condition effect nor a genotype-by-condition interaction effect. While the modes of muscular strength testing are different, and this study is the first to assess 1RM changes regarding caffeine and genotype interactions, the findings from the present study support previous research showing that acute changes in muscular strength do not appear to be impacted by CYP1A2 genotypes [1,14,15,16,22].

Muscular endurance was assessed by performing RTF at 70% of participants’ leg press and bench press 1RM. As previously stated, the main effect observed in leg press RTF for caffeine indicated that CAF had a higher RTF. Additionally, the observed main effect for genotype indicated the FAST group was able to perform more repetitions to failure compared to the SLOW group. This supports previous research in males [2], which reported similar findings in the repetitions to failure of bench press, leg press, seated cable row, and shoulder press at 85% of their 1RM with the same dose of CAF at 6 mg/kg bw. Spineli and colleagues [22] also administered a 6 mg/kg dose of CAF to young males and females and observed an increase in the number of RTF in the muscular endurance exercise testing protocols, including sit-ups and push-ups in one minute; however, there was no main effect revealed between genotypes. Another study [14] reported similar findings in males that caffeine improved performance in muscular endurance consisting of repetitions to failure for bench press at multiple loads (25–90% of 1RM) after a 3 mg/kg bw dose, but without a main effect for genotype groups, which they proposed may be due to the low dose of caffeine or low sample size. The findings from the present study corroborate the published literature on repetitions to failure in the leg press exercise, but not the bench press exercise as there was no condition or genotype main effect for the latter [2,14,22].

Anaerobic power was determined by assessing peak and mean power output in watts from the Wingate 30 s cycle test. There were no main effects or significant differences for condition or genotype revealed from the statistical analysis. Similar to the design of the present study, prior research assessed anaerobic power by using the 30 s Wingate cycle test for peak and mean power output as the last performance measure at the end of each testing session [1,14]. The Wingate cycle test followed a bout of bench press repetitions to failure and a countermovement jump test for the men in one study [14], while the males and females in the other study completed the Wingate cycle test after a cognitive visual attention test [1]. Grgic and colleagues (2020) observed a main effect for caffeine overall in the improvement in peak and mean power after a 3 mg/kg bw dose without a main effect for genotypes [14]. Salinero and researchers [1] also used a 3 mg/kg bw dose of caffeine and observed a main effect for caffeine with just peak power, with no main effects for genotypes. These findings are contradictory to the findings of the present study, which indicate that administering a moderate caffeine dose of 6 mg/kg bw did not induce a statistically significant difference in performance. Although the Wingate mean power output condition-by-genotype interaction did not reach statistical significance (*p* = 0.093), the observed effect size (partial eta-squared = 0.083) suggests a potential for a meaningful interaction. Similarly, although not statistically significant (*p* = 0.056), the Wingate mean power output data indicate a 11.6% difference between the SLOW (420.06 W) and FAST (374.00 W) groups following caffeine ingestion. This suggests a possible influence of genotype on caffeine’s ergogenic effects with implications for performance testing order during time-restricted sessions. Prior research suggests differences in caffeine metabolism rates between FAST and SLOW genotypes [1]. This, combined with our chosen testing order, could be relevant to these findings. The FAST group’s higher performance in LPRTF (early in the experimental testing session) coupled with the SLOW group’s near-significant improvement in the Wingate test (last element of the experimental testing session) aligns with this influence of the SLOW vs. FAST genotype. Interestingly, caffeine did not significantly influence bench press performance in either genotype. This could be due to several factors, including the placement of the bench press exercises in the middle of the testing session, which might have been outside of the peak ergogenic window for both the FAST and SLOW groups, potentially explaining the lack of observed benefit. This should be explored in the future using serum caffeine measures to detect pharmacokinetic differences between genotypes and the subsequent effect on performance. Thus, although these results did not reach significance, they present the idea that there is potential to reach a level of statistical significance with a greater sample size and exploration. The exact mechanism for this observed difference is still unclear, and thus warrants continued research on power output between genotype groups after acute caffeine ingestion.

It is widely known that subjective mood states, such as energy, concentration, and alertness, can improve after ingesting a range of caffeine doses; however, caffeine has also shown to produce undesirable effects, such as tachycardia, heart palpitations, anxiety, nervousness, and jitteriness [22,23]. In the present study, subjective outcomes, including subjective index score and follow-up assessment measures, were mixed. The FAST group had a higher overall subjective index score after ingesting 6 mg/kg bw caffeine, which supports previous research indicating that low to moderate doses of caffeine can improve mood states such as alertness and energy [24]. Interestingly, the SLOW group experienced more dizziness after ingesting the moderate caffeine dose, which supports the consensus that caffeine can result in acute adverse effects [25]. Moreover, the follow-up assessment was filled out after the last performance measure (Wingate cycle test) was completed, and due to the high-intensity nature of the test, the spike in dizziness may be attributable to the effects of exercise and the Wingate cycle test. Previous research has explored other subjective effects of caffeine [26] without factoring in genotypes. These findings reinforce the established link between caffeine ingestion and subjective responses; however, the literature identifying differences between CYP1A2 genotypes after ingesting caffeine in terms of the impact on subjective measures is lacking in general and must be expanded further.

This study presents some limitations that may provide insight to the outcome measures. First, the participants were instructed to arrive at the lab in a 12 h fasted state. If the participants were not accustomed to performing strength and power tests in a fasted state, this may have impeded their performance or resulted in adverse effects. Second, the sample size (n = 36) did not reach the threshold calculated by G*Power (n = 42) to reach adequate power due to subjects dropping out of the study. A greater sample size would allow for better generalizations about each genotype and their performance outcomes with this specific study design. Finally, the participants were required to complete all testing within 2 h due to time constraints. This time restriction imposes practical challenges and limitations on the ability to determine genotypical differences within the allotted time for data collection. Despite these limitations, this investigation provides insight and added information regarding the impact that CYP1A2 genotypes have on the ergogenic effects of caffeine on resistance exercise performance in resistance-trained females.

## 5. Conclusions

The CYP1A2 genotype did not significantly impact ergogenic responses in most physical performance measures following acute moderate caffeine intake in this female population. However, participant-reported subjective measures did differ between conditions and genotype groups. The outcomes of this investigation suggest that while CYP1A2 genotypes may impact the ergogenic effects of caffeine to some degree, there may be other factors influencing physical performance and physiological response. This study underscores the need for further research to elucidate the variable pharmacokinetic properties of caffeine and the extent to which CYP1A2 influences interindividual variability in metabolism rates and exercise performance, particularly in females.

## Figures and Tables

**Table 1:** Descriptive characteristics of participants by CYP1A2 genotype.

Characteristic	FAST (n = 20)	SLOW (n = 16)
Age (yrs)	21.6 ± 2.2	22.3 ± 3.8
Height (cm)	163.2 ± 9.6	165.6 ± 5.3
Weight (kg)	70.3 ± 15.4	68.7 ± 14.3
Lean Body Mass (kg)	45.2 ± 8.3	43.4 ± 7.2
Fat Mass (kg)	20.5 ± 8.9	20.4 ± 9.7
Percent Body Fat (%)	29.4 ± 7.1	29.9 ± 7.2

Descriptive characteristics presented as means ± standard deviations separated into two genotype groups (FAST; SLOW) as determined at the FAM session.

**Table 2:** Side effects follow-up assessment.

Follow-Up Variable	CAF	PLA	*p*-Value
Dizziness	0.76 ± 1.075	0.35 ± 0.812	0.003 *
Headache	0.44 ± 0.991	0.35 ± 0.774	0.608
Fast/Racing Heart Rate	1.79 ± 1.338	1.74 ± 1.421	0.754
Heart Skipping/Palpitations	0.24 ± 0.699	0.15 ± 0.558	0.586
Shortness of Breath	1.32 ± 1.364	1.26 ± 1.483	0.494
Nervousness	0.59 ± 1.048	0.35 ± 0.849	0.304
Blurred Vision	0.26 ± 0.618	0.09 ± 0.288	0.084

Side effects assessment data are presented in means ± standard deviations and *p*-values between treatment groups (CAF vs. PLA). * denotes significant difference between conditions (CAF vs. PLA).

## Data Availability

The original contributions presented in the study are included in the article, further inquiries can be directed to the corresponding authors.
